# Reconstructing and analyzing the invariances of low‐dose CT image denoising networks

**DOI:** 10.1002/mp.17413

**Published:** 2024-09-30

**Authors:** Elias Eulig, Fabian Jäger, Joscha Maier, Björn Ommer, Marc Kachelrieß

**Affiliations:** ^1^ Division of X‐Ray Imaging and Computed Tomography German Cancer Research Center (DKFZ) Heidelberg Germany; ^2^ Faculty of Physics and Astronomy Heidelberg University Heidelberg Germany; ^3^ CompVis @ LMU Munich and MCML Munich Germany; ^4^ Medical Faculty Heidelberg Heidelberg University Heidelberg Germany

**Keywords:** computed tomography, deep learning, explainability, invariances, low‐dose, robustness

## Abstract

**Background:**

Deep learning‐based methods led to significant advancements in many areas of medical imaging, most of which are concerned with the reduction of artifacts caused by motion, scatter, or noise. However, with most neural networks being black boxes, they remain notoriously difficult to interpret, hindering their clinical implementation. In particular, it has been shown that networks exhibit invariances w.r.t. input features, that is, they learn to ignore certain information in the input data.

**Purpose:**

To improve the interpretability of deep learning‐based low‐dose CT image denoising networks.

**Methods:**

We learn a complete data representation of low‐dose input images using a conditional variational autoencoder (cVAE). In this representation, invariances of any given denoising network are then disentangled from the information it is not invariant to using a conditional invertible neural network (cINN). At test time, image‐space invariances are generated by applying the inverse of the cINN and subsequent decoding using the cVAE. We propose two methods to analyze sampled invariances and to find those that correspond to alterations of anatomical structures.

**Results:**

The proposed method is applied to four popular deep learning‐based low‐dose CT image denoising networks. We find that the networks are not only invariant to noise amplitude and realizations, but also to anatomical structures.

**Conclusions:**

The proposed method is capable of reconstructing and analyzing invariances of deep learning‐based low‐dose CT image denoising networks. This is an important step toward interpreting deep learning‐based methods for medical imaging, which is essential for their clinical implementation.

## 1 INTRODUCTION

Deep learning‐based methods have revolutionized the field of medical image formation in general and computed tomography (CT) in particular by delivering cutting‐edge solutions to a wide range of problems. These include noise reduction,^1^, ^2^, ^3^, ^4^, ^5^ image reconstruction,^6^, ^7^, ^8^ scatter estimation,^9^, ^10^, ^11^ and artifact reduction.^12^, ^13^ Most of these problems, however, are not injective, meaning that a single target‐domain (e.g., artifact‐free) image can be derived from different source‐domain (e.g., artifact‐deteriorated) images. Therefore, a good network for these tasks *must* be invariant to some input features (e.g., image noise for low‐dose reconstruction) to some extent.^14^ From a network architecture perspective, invariances can be realized by certain noninjective layers such as max‐pooling layers or convolutions with certain weight configurations.

In this study, we aim to investigate and interpret these invariances in low‐dose computed tomography (LDCT) image denoising networks — a prevalent application of deep learning in CT image formation. Such an analysis can provide valuable insights into the networks behavior and help in identifying potential biases or shortcomings of the networks and their training data. This is important in order to improve the interpretability and robustness of deep learning‐based methods for medical imaging, which is an essential step toward bridging the implementation gap of deep learning‐based methods in medical imaging.^15^, ^16^

### 1.1 Deep learning‐based low‐dose CT image denoising

While our method for reconstructing and analyzing invariances of image‐to‐image translation networks is applicable to a wide range of deep learning‐based applications for CT and other modalities, we here focus on the task of LDCT due to the abundance of publications in the field* and the availability of open‐source datasets.

LDCT aims at providing an image x with a lower dose than conventional CT acquisitions, which is typically accomplished by decreasing the tube current and consequently reducing the x‐ray flux. However, this approach increases noise in the projection data due to photon starvation. As a result, when these images are reconstructed using standard filtered back projection (FBP), they exhibit unwanted noise and streak artifacts, potentially reducing diagnostic value.

To mitigate these artifacts, advanced reconstruction techniques such as iterative reconstruction can be employed. These methods effectively suppress the artifacts but are computationally expensive, often limiting their clinical applicability in time‐critical scenarios, such as emergency rooms. On the other hand, denoising methods present a computationally efficient solution and can be integrated seamlessly into any existing reconstruction pipeline. These algorithms may be conventional,^17^, ^18^, ^19^, ^20^ or data‐driven^1^, ^2^, ^21^, ^22^, ^23^ and can be applied in either projection domain, image domain, or both. Particularly, deep learning‐based methods applied to reconstructed images are prevalent in the literature since they do not require access to the (often proprietary) projection data.

Deep learning‐based image domain denoising methods usually learn a mapping $f_{\theta }:x^{\left(i\right)}\to y^{\left(i\right)}$ from low‐dose images $x^{\left(i\right)}$ (i.e., images reconstructed from low‐dose projections via FBP) to high‐dose images $y^{\left(i\right)}$, where $f_{\theta }$ is a deep neural network (DNN) with parameters θ. Most methods optimize the parameters in a supervised fashion by minimizing some (typically pixel‐wise) loss L over the training set ${\left\{\left(x^{\left(i\right)},y^{\left(i\right)}\right)\right\}}_{i=1}^{N}$

(1) $$\begin{array}{c} \theta^{*}=\underset{\theta }{\arg\min}\frac{1}{N}\sum_{i=1}^{N}L\left(f_{\theta }\left(x^{\left(i\right)}\right),y^{\left(i\right)}\right)\;. \end{array}$$

Numerous other works train $f_{\theta }$ unsupervised or self‐supervised. These include methods leveraging the image prior of convolutional neural networks (CNNs),^8^ intrinsic similarities within the training data (e.g., across views or patches),^5^, ^24^, ^25^, ^26^, ^27^ or methods from deep metric learning (DML).^28^ We refer the reader to Lei et al., 2024^29^ for a comprehensive review of these methods.

For a fair comparison between denoising algorithms, we henceforth focus on methods trained using Equation 1 that vary in their architectural design of $f_{\theta }$ and the choice of L used for learning the parameters θ.

### 1.2 Reconstructing invariances of DNNs

Previously, Rombach et al.^14^ presented a method to reconstruct the invariances of some image classification network $f:R^{n\times m}\to {\left\{0,1\right\}}^{c}$, with n×m being the image size and c the number of classes, using conditional invertible neural networks (cINNs). Let $\bar{z}\in R^{d}$ denote any internal latent representation (e.g., if d=n×m×64 this could be the output of a zero‐padded convolutional layer with 64 filters) that we can get by decomposing f into $f\left(x\right)=\Psi \left(\bar{z}\right)=\Psi \left(\Phi \left(x\right)\right)$, where $\Phi :R^{n\times m}\to R^{d}$ and $\Psi :R^{d}\to {\left\{0,1\right\}}^{c}$. To then find out which information about x is captured in $\bar{z}$ and which is missing (i.e., the invariances of Φ), we need a compact data representation of x. The authors propose to learn such a data representation z by training a variational autoencoder (VAE) comprised of an encoder E and decoder D. Since z=E(x) now not only contains the information of x that is captured in $\bar{z}$, but also ϕ’s invariances v, we need to disentangle these two components. This is achieved by training a normalizing flow $t(\cdot |\bar{z}):z\to v$ that maps between those two domains, conditioned on the network representation $\bar{z}$. Since t is invertible, we can then sample from p(v) (here assumed to be normal) and apply $t^{-1}$ to obtain samples from p(z). Finally, we can reconstruct the invariances of Φ in image space by applying the pretrained decoder D to the samples z.

This method has later been adapted to reconstruct the invariances of CT image denoising networks.^30^ However, due to the fact that LDCT denoising networks exhibit fewer and more subtle invariances than image classification networks, some reconstructed invariances may be attributed to the VAE rather than the denoising networks. This is further exacerbated by the diversity of medical image data, which makes it difficult for the VAE to learn an almost complete data representation of the input data. In this work, we propose to reconstruct the invariances of LDCT denoising networks by training a *conditional* VAE, therefore improving its data representation compared to previous works. We also investigate the invariances of more recent and advanced denoising networks and introduce methods to analyze the sampled invariances.†

## 2 METHODS

In the following we will present a method to sample and analyze the invariances of LDCT denoising networks. Note that the method presented herein is network and application‐agnostic and therefore potentially applicable to many other image‐to‐image translation tasks in medical imaging such as metal artifact correction or sparse view CT.

### 2.1 Dataset

For all our experiments, we use the 50 chest exams provided in the open‐source *Low‐dose CT Image and Projection Dataset*.^31^ For each scan in the dataset, the authors simulated low‐dose reconstructions by inserting noise in the projection domain. These reconstructions correspond to a dose level of 10%.

We randomly split the respective acquisitions (on a patient level) into 70% training (35 patients), 20%validation (10 patients), and 10% (5 patients) test data. During training and validation, we employ a weighted sampling scheme, ensuring that every acquisition has an equal probability of being selected, regardless of the varying number of slices per acquisition. All data are normalized to have zero‐mean, unit‐variance before feeding them to the networks.

### 2.2 Denoising methods

We reconstruct the invariances of four different deep learning‐based image denoising algorithms, which are summarized in the following. We refer the reader to the respective publications for more details.

**CNN‐10** ^1^ One of the earliest deep learning‐based methods for LDCT image denoising. The authors propose a simple three‐layer CNN which receives low‐dose images as input and is trained using Equation 1 with the mean‐squared‐error loss.

**RED‐CNN** ^2^ This method builds upon CNN‐10 by incorporating a deeper residual encoder‐decoder architecture but keeps the overall training procedure identical. In previous works,^32^ it has been shown that this method outperforms many other (and notably newer) deep learning‐based denoising methods.

**WGAN‐VGG** ^3^ The authors improve on CNN‐10 by using a deeper network architecture and by training it together with a convolutional critic as Wasserstein‐GAN.^33^ Furthermore, they added a perceptual loss^34^ derived from a pretrained VGG to the overall generator loss. In comparison with traditional pixel‐wise loss functions, this approach leads to denoised samples that exhibit more refined details and authentic noise textures.

**DU‐GAN** ^22^ Similar to WGAN‐VGG, the authors employ an adversarial training scheme, but use a U‐Net‐based discriminator^35^ which allows for per‐pixel feedback to the generator network, for which they use the same structure as RED‐CNN.

All four methods are trained using the data as described in Section 2.1 and we use the best performing network on the validation data for subsequent invariance reconstruction. Additional training specifics for each method are provided in Supplementary Materials A.1.

### 2.3 Reconstructing invariances

**FIGURE 1:**
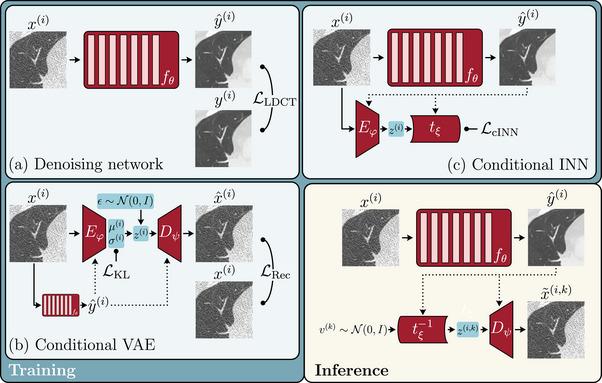
Overview of our method to reconstruct invariances of LDCT image denoising networks. Solid arrows represent inputs/outputs to modules, dotted arrows represent conditional inputs to a module. We indicate points where loss functions L are calculated using 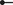 L. Training: (a) training of the denoising network $f_{\theta }$ using low‐dose images $x^{\left(i\right)}$ and corresponding high‐dose images $y^{\left(i\right)}$. $L_{\text{LDCT}}$ can be some pixel‐wise or adversarial loss, or a combination of both (Equation 1); (b) training of the conditional VAE with encoder $E_{\varphi }$ and decoder $D_{\psi }$ conditioned on the denoised images ${\hat{y}}^{\left(i\right)}=f_{\theta }\left(x^{\left(i\right)}\right)$. $L_{\text{KL}}$ and $L_{\text{Rec}}$ are the Kullback–Leibler divergence and reconstruction loss, respectively (Equation 4); and (c) training of the conditional INN $t_{\xi }$ to disentangle the invariances of the denoising network $f_{\theta }$ from the latent representation $z^{\left(i\right)}$ learned by the VAE. $L_{\text{cINN}}$ is the loss function of the cINN (Equation 6). Inference: We sample new invariances $v^{\left(k\right)}$ from the Gaussian distribution of invariances and apply the inverse cINN to obtain samples $z^{\left(i,k\right)}$. We then decode the samples using the conditional decoder $D_{\psi }$ to obtain the invariance reconstructions ${\tilde{x}}^{\left(i,k\right)}$ (Section 2.3).

Our pipeline to reconstruct invariances (Figure 1) comprises three components:

(a) The LDCT denoising network $f_{\theta }$, that receives low‐dose images $x^{\left(i\right)}$ as input and predicts high‐dose images ${\hat{y}}^{\left(i\right)}=f_{\theta }\left(x^{\left(i\right)}\right)$ (Section 2.2).
(b) A conditional VAE $D_{\psi }\circ E_{\varphi }$ that is trained to learn a complete data representation $z\in R^{M}$ of the low‐dose images. We condition both encoder $E_{\varphi }$ and decoder $D_{\psi }$ on predictions of the denoising network ${\hat{y}}^{\left(i\right)}$, thereby improving their encoding/reconstruction capabilities (Section 2.3.1).
(c) A cINN that disentangles the information in z that the denoising network $f_{\theta }$ is invariant to from the one it is not invariant to. To reconstruct invariances, we then sample from the Gaussian distribution of invariances, apply the inverse cINN, and decode the samples using the (fixed) conditional decoder.

#### 2.3.1 Training of the conditional VAE

In order to reconstruct which information of low‐dose images a given denoising network $f_{\theta }$ has learned to represent and which to ignore (i.e., its invariances), we first need to learn an (almost) complete representation of low‐dose images $x^{\left(i\right)}$. We do so by training a conditional variational autoencoder comprised of a conditional probabilistic encoder $E_{\varphi }$ defining the distribution $q_{\varphi }\left(z|x,\hat{y}\right)$ and conditional probabilistic decoder $D_{\psi }$ defining $p_{\psi }\left(x|z,\hat{y}\right)$. We assume a Gaussian prior $p(z|\hat{y})$ on latent variables z and approximate the posterior with a Gaussian $q_{\varphi }\left(z|x,\hat{y}\right)$ with diagonal covariance. Let $\mu^{\left(i\right)},\sigma^{\left(i\right)}\in R^{M}$ denote the mean and standard deviation predicted by the encoder $E_{\varphi }$ for the i‐th sample $x^{\left(i\right)}$, conditioned on its respective denoised image ${\hat{y}}^{\left(i\right)}$. Then,

(2) $$\begin{array}{c} \ln q_{\varphi }\left(z|x^{\left(i\right)},{\hat{y}}^{\left(i\right)}\right)=\ln N\left(z;\mu^{\left(i\right)},\mathrm{diag}\left(\sigma^{\left(i\right)}\right)\right)\;. \end{array}$$

As for any variational autoencoder,^36^ both encoder and decoder are trained to maximize the expectation $E_{i}\left[\text{ELBO}(x^{\left(i\right)},{\hat{y}}^{\left(i\right)})\right]$ with the evidence lower bound (ELBO) being

(3) $$\begin{array}{ccc} \text{ELBO}(x^{\left(i\right)},{\hat{y}}^{\left(i\right)}) & = & E_{z∼q_{\varphi }\left(z|x^{\left(i\right)},{\hat{y}}^{\left(i\right)}\right)}\left[\ln p_{\psi }\left(x^{\left(i\right)}|z,{\hat{y}}^{\left(i\right)}\right)\right]\\ & & -D_{\text{KL}}\left(q_{\varphi }\left(z|x^{\left(i\right)},{\hat{y}}^{\left(i\right)}\right)\left|\right|p_{\psi }\left(z|{\hat{y}}^{\left(i\right)}\right)\right)\;, \end{array}$$

where $D_{\text{KL}}\left(q||p\right)$ denotes the Kullback–Leibler (KL) divergence between distributions q,p. Using the fact that the KL divergence between two Gaussians can be computed analytically, we derive the loss function

(4) LVAE(φ,ψ)=−Ei∑m=1Mlnpψ(x(i)|z(i),y^(i))+12∑m=1M1+ln(σm(i))2−(μm(i))2−(σm(i))2=−Ei∥x(i)−x^(i)∥︸LRec+12∑m=1M1+ln(σm(i))2−(μm(i))2−(σm(i))2︸LKL,

where M=dim(z), ${\hat{x}}^{\left(i\right)}=D_{\psi }\left(\mu^{\left(i\right)}+\sigma^{\left(i\right)}\varepsilon |{\hat{y}}^{\left(i\right)}\right)$ with ε∼N(0,I).

Conditioning the VAE on auxiliary information^37^ eases the task for both encoder and decoder, as they can focus on the information about the input image that is not contained in the auxiliary information (here: the denoised image $\hat{y}$) already‡.

In our experiments, both $E_{\varphi }$ and $D_{\psi }$ are parameterized by DNNs, with $E_{\varphi }$ being an ImageNet‐pretrained ResNet‐50^38^ and $D_{\psi }$ based on BigGAN.^39^ To improve reconstruction quality, we use a perceptual loss^34^ and adversarial loss in addition to the pixel‐wise loss in Equation 4. We refer the reader to Supplementary Material A.2 for more details on the training procedure. For comparison, we also train a VAE without the conditioning on $\hat{y}$ (as explored in previous works^14^, ^30^), but otherwise identical architecture and training procedure.

#### 2.3.2 Training of the conditional invertible neural network

The latent representation z does not only contain invariances of the denoised image ${\hat{y}}^{\left(i\right)}$ but also information about the input image x. Therefore, we need to disentangle these two components, that is, extract the invariances v of the denoising networks' prediction ${\hat{y}}^{\left(i\right)}$ from the other information in z. Thus, we need to learn a mapping $t_{\xi }$ from z to some space of invariances, *given* a denoised image ${\hat{y}}^{\left(i\right)}$. Let this space of invariances p(v) be a standard Gaussian distribution, that is, p(v)∼N(0,I). Then, $t_{\xi }:p\left(z|\hat{y}\right)\to p\left(v\right)$ allows us to generate $v^{\left(i\right)}=t_{\xi }\left(z^{\left(i\right)}|{\hat{y}}^{\left(i\right)}\right)$ for any given sample i. In our experiments, $t_{\xi }$ is realized by a conditional invertible neural network with parameters ξ, that is, a normalizing flow conditioned on ${\hat{y}}^{\left(i\right)}$.^40^, ^41^, ^42^, ^43^

As for any cINN,^43^ we can find optimal parameters ξ via standard maximum likelihood training. Using the change‐of‐variables formula gives us the likelihood

(5) $$\begin{array}{c} q\left(z^{\left(i\right)}|{\hat{y}}^{\left(i\right)},\xi \right)=p\left(t_{\xi }\left(z^{\left(i\right)}|{\hat{y}}^{\left(i\right)}\right)\right)\left|J^{\left(i\right)}\right|\;, \end{array}$$

with $J^{\left(i\right)}=\det\left(\frac{\partial t_{\xi }\left(z^{\left(i\right)}|{\hat{y}}^{\left(i\right)}\right)}{\partial z^{\left(i\right)}}\right)$. The loss function over training samples i then reads as

(6) $$\begin{array}{cc} L_{\text{cINN}} & =E_{i}\left[-\ln q(z^{\left(i\right)}|{\hat{y}}^{\left(i\right)},\xi )\right]\\ & =E_{i}\left[-\underset{\ell (t_{\xi }\left(z^{\left(i\right)}|{\hat{y}}^{\left(i\right)}\right);0,1)}{\underset{︸}{\ln p\left(t_{\xi }\left(z^{\left(i\right)}|{\hat{y}}^{\left(i\right)}\right)\right)}}-\ln\left|J^{\left(i\right)}\right|\right]\\ & =E_{i}\left[\frac{1}{2}{∥t_{\xi }\left(z^{\left(i\right)}|{\hat{y}}^{\left(i\right)}\right)∥}_{2}^{2}-\ln\left|J^{\left(i\right)}\right|\right]\;, \end{array}$$

where in the last step we used the log‐likelihood over samples $x=\left\{x_{1},x_{2},\cdots ,x_{N}\right\}$ of a standard Gaussian distribution $\ell \left(x;\mu ,\sigma^{2}\right)=-N\ln\sigma -\frac{N}{2}\ln\left(2\pi \right)-\frac{1}{2\sigma^{2}}{∥x-\mu ∥}_{2}^{2}$ and the assumption that p(v) is a normal distribution with zero mean and unit variance. The first line in Equation 6 is the log‐likelihood of observing some representation $z^{\left(i\right)}$ given the corresponding denoised image ${\hat{y}}^{\left(i\right)}$ under parameters ξ. After optimization of parameters ξ, we can sample from p(v) and apply the inverse $t_{\xi }^{-1}$ to map invariances to the input data representation, conditioned on the denoised image ${\hat{y}}^{\left(i\right)}$. We refer the reader to Supplementary Material A.3 for more details on the architecture and training of $t_{\xi }$.

#### 2.3.3 Sampling invariances

Once conditional VAE and cINN are trained, we can generate invariance samples ${\tilde{x}}^{\left(i,k\right)}$ for a given sample $x^{\left(i\right)}$ from the test set and a (trained) denoising network $f_{\theta }$ as follows:

1. Denoise the image using the pretrained denoising network $f_{\theta }$: ${\hat{y}}^{\left(i\right)}=f_{\theta }\left(x^{\left(i\right)}\right)$.
2. Sample $v^{\left(k\right)}∼N\left(0,I\right)$ from the space of invariances.
3. Apply the inverse of the cINN $t_{\xi }^{-1}$ to the sampled invariance: $z^{\left(i,k\right)}=t_{\xi }^{-1}\left(v^{\left(k\right)}|{\hat{y}}^{\left(i\right)}\right)$.
4. Decode the samples using the (fixed) conditional decoder $D_{\psi }$ to obtain the invariance reconstructions ${\tilde{x}}^{\left(i,k\right)}=D_{\psi }\left(z^{\left(i,k\right)}|{\hat{y}}^{\left(i\right)}\right)$.

Every ${\tilde{x}}^{\left(i,k\right)}$ is then a sample from the distribution of invariances of the denoising network $f_{\theta }$ for the $i^{\mathrm{th}}$ low‐dose image $x^{\left(i\right)}$ and two images ${\tilde{x}}^{\left(i,k\right)},{\tilde{x}}^{\left(i,l\right)}$ differ only in their realization of invariances.

### 2.4 Analyzing invariances

**FIGURE 2:**
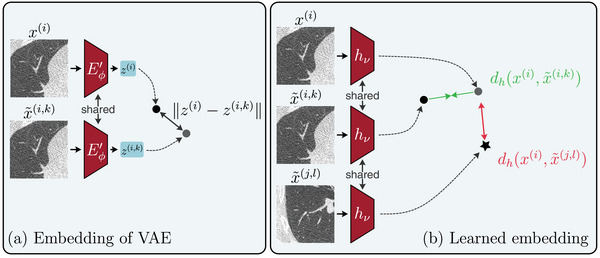
Overview of our methods to analyze sampled invariances. (a) based on the embedding of an unconditional VAE, with encoder $E_{\phi }^{\prime }$, whose latent space is dominated by content‐related information. Applying the same encoder to the input image $x^{\left(i\right)}$ and sampled invariance ${\tilde{x}}^{\left(i,k\right)}$, we can measure the content similarity between the two and (b) based on a learned embedding which is trained with a triplet loss (Equation 8) to map low‐dose images $x^{\left(i\right)}$ closer to invariance samples ${\tilde{x}}^{\left(i,k\right)}$ corresponding to the same sample i than to invariance samples ${\tilde{x}}^{\left(j,l\right)},j\neq i$.

In our experiments, we find that the most prominent invariances of LDCT denoising networks are related to the noise level and noise realization of the input images. While this is expected and a desirable property of any denoising algorithm, this does not answer our initial question of whether LDCT denoising networks are invariant to anatomical structures or other image *content*. Finding such differences in the pixel space is challenging, as differences in noise realizations can easily overshadow differences in content. We therefore propose to analyze the invariances in an embedding space instead and compare two different methods to do so (Figure 2). The first is based on an embedding learned by an unconditional VAE (Sections 2.4.1, and Figure 2a). The second is based on a learned embedding of the invariances using a DML approach (Sections 2.4.2, and Figure 2b).

#### 2.4.1 Using an unconditional VAE

The conditional VAE is trained to learn a complete representation of the low‐dose images, which includes both their noise level and noise realization as well as the anatomical structures and other image content. However, since for the conditional VAE, the latent space follows the distribution $p(z|{\hat{y}}^{\left(i\right)})$, we cannot compare different samples i with another. Instead, we use an unconditional VAE for which p(z) is standard Gaussian distributed but which is otherwise identical to the conditional one. Since noise is generally harder to model than content, we expect the learned representation $E_{\phi }^{\prime }\left(x^{\left(i\right)}\right)$ to be dominated by content‐related information.

We can then use differences in the latent space as a proxy for differences in anatomical content between invariance samples ${\tilde{x}}^{\left(i,k\right)}$ and low‐dose inputs $x^{\left(i\right)}$. To this end we compute the cosine similarity between the latent representations of the low‐dose input and invariance samples $z^{\left(i\right)}=E_{\phi }^{\prime }\left(x^{\left(i\right)}\right),\;z^{\left(i,k\right)}=E_{\phi }^{\prime }\left({\tilde{x}}^{\left(i,k\right)}\right)$ as

(7) $$\cos\left(z^{\left(i\right)},z^{\left(i,k\right)}\right)=\frac{z^{\left(i\right)}\cdot z^{\left(i,k\right)}}{∥z^{\left(i\right)}∥∥z^{\left(i,k\right)}∥}.$$

#### 2.4.2 Using a learned embedding

We can also learn an embedding of the invariances using a DML approach. Metric learning generally seeks to learn a metric function h such that semantic relations between datapoints $x^{\left(i\right)},x^{\left(j\right)}\in X$ are depicted by metric distances $d_{h}\left(x^{\left(i\right)},x^{\left(j\right)}\right):=d\left(h\left(x^{\left(i\right)}\right),h\left(x^{\left(j\right)}\right)\right)$, with d(·,·) being some distance, in the embedding h(·). In DML, h is typically parameterized by a deep neural network $h_{\nu }:=h$ with weights ν being learned by minimizing a loss function that encourages the network to map similar (w.r.t. some semantic relation) samples closer together than dissimilar ones. To do so, many different loss functions have been proposed, most popularly ranking‐based loss functions.^44^, ^45^, ^46^ We refer the reader to Roth et al., 2020^47^ for a nice overview of training strategies in DML.

In our experiments, we use the triplet loss^45^ to learn an embedding in which low‐dose inputs $x^{\left(i\right)}$ are closer to invariance samples ${\tilde{x}}^{\left(i,k\right)},\forall k$ corresponding to the same sample i (with same anatomy) than to invariances samples $x^{\left(j,l\right)},\forall l,j\neq i$ corresponding to different samples (with different anatomy). The loss function for $h_{\nu }$ then reads as

(8) $$\begin{array}{c} L_{\text{Triplet}}=E_{i,j}{\left[d_{h}\left(x^{\left(i\right)},{\tilde{x}}^{\left(i,k\right)}\right)-d_{h}\left(x^{\left(i\right)},{\tilde{x}}^{\left(j,l\right)}\right)+\alpha \right]}_{+}\;, \end{array}$$

with α being some prespecified margin. In our experiments, we use a pretrained ResNet‐50^38^ as $h_{\nu }$ and select triplets $\left(a,p,n\right):=\left(x^{\left(i\right)},{\tilde{x}}^{\left(i,k\right)},{\tilde{x}}^{\left(j,l\right)}\right)$ using the semi‐hard triplet mining strategy.^45^ We refer the reader to Supplementary Material A.4 for more details on the training procedure.

## 3 RESULTS

### 3.1 Denoising of LDCT images

**FIGURE 3:**
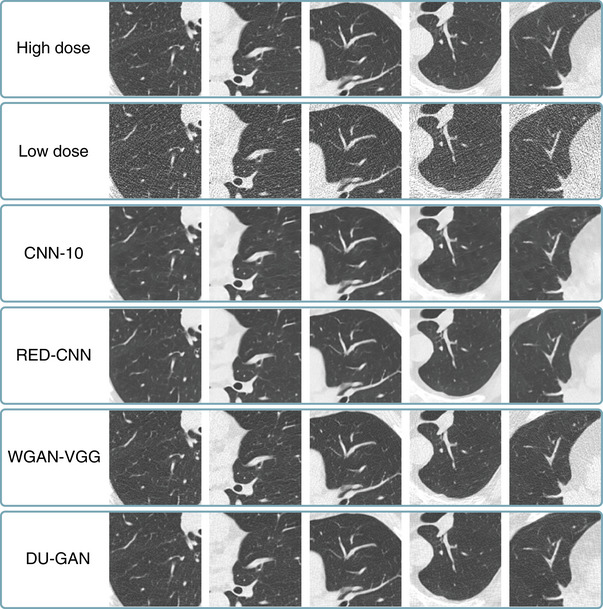
High‐dose, low‐dose, and denoising results for the four methods described in Section 2.2. We show results for random axial slices and crops of size 128×128px for all five patients from the test set. Center (C) and width (W) are C=−600HU, W=1500HU.

We first verify qualitatively that the denoising methods (compare Section 2.2) perform as expected and are able to denoise LDCT images similarly as reported in their respective publications. To this end, we show results for random axial slices of all five test patients in Figure 3. For each patient of the test set, we show the high‐dose image, the low‐dose image, and the respective denoised images. While all methods are able to reduce noise and streak artifacts compared to the low‐dose image, the results of WGAN‐VGG^3^ and DU‐GAN^22^ show more realistic noise structures and exhibit finer details compared to the two methods trained using a pixel‐wise loss exclusively. This is in line with the findings of Yang et al.^3^ and Huang et al.^22^ and can be attributed to the additional perceptual loss (for WGAN‐VGG) and adversarial loss (for both WGAN‐VGG and DU‐GAN).

Upon quantitative evaluation (Table 1), we find that RED‐CNN performs best in terms of the structural similarity index measure (SSIM), peak signal‐to‐noise ratio (PSNR), and root‐mean‐square error (RMSE). However, it is important to note that these metrics do not correlate well with human reader ratings (the gold standard in terms of medical image quality assessment) for computed tomography.^48^, ^49^, ^50^ Since this work is not concerned with the evaluation of the denoising methods themselves, but rather with their invariances, we do not further investigate the performance of the denoising methods and leave the development of better metrics for future work.

**TABLE 1:** Quantitative evaluation of the denoising methods described in Section 2.2.

	SSIM	PSNR (dB)	RMSE (HU)
LD	0.312 ± 0.072	18.1 ± 2.5	236 ± 86
CNN‐10	0.56 ± 0.10	27.3 ± 2.1	72 ± 19
RED‐CNN	**0.58 ± 0.10**	**28.0 ± 2.2**	**66 ± 18**
WGAN‐VGG	0.505 ± 0.099	25.3 ± 2.2	91 ± 26
DU‐GAN	0.544 ± 0.096	26.3 ± 2.2	80 ± 22

*Note*: We report the mean and standard deviation of the SSIM, PSNR, and RMSE over all axial slices of the test set. **Bold** values highlight the best performing method for each metric.

### 3.2 VAE reconstructions

**FIGURE 4:**
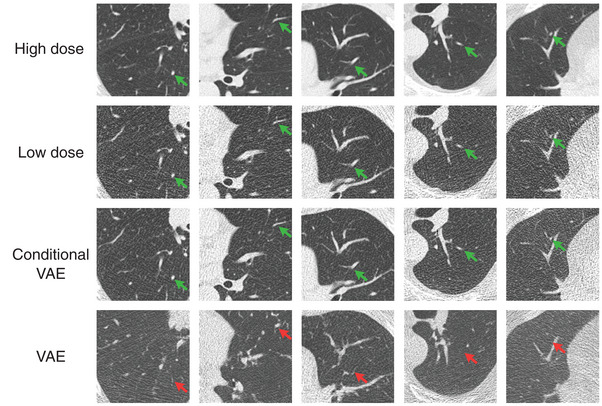
High‐dose, low‐dose, and VAE reconstructions for the conditional VAE (here conditioned on RED‐CNN) described in Section 2.3.1. Patients, axial slices and crops correspond to those shown in Figure 3. Additionally, we show the reconstructions of an unconditional VAE (as used in previous work^30^) for comparison. C=−600HU, W=1500HU.

Next, we evaluate the reconstruction capabilities of the conditional VAE (Section 2.3.1) for random axial slices of all patients of the test set in Figure 4. We find that reconstructions of the conditional VAE (Figure 4; third row) are very similar to the input low‐dose images (Figure 4; second row) for all exam types. Additionally, we show the reconstructions of an unconditional VAE (Figure 4; last row) as it was used in previous work to reconstruct invariances of LDCT denoising networks^30^ for comparison. While the unconditional VAE is able to generate realistic low‐dose images that reflect, to some extent, the anatomical structures of the low‐dose input images, it fails to capture fine details and removes or hallucinates many of the anatomical structures in the reconstructions (compare red arrows in Figure 4). We show reconstruction results for all conditional VAEs (conditioned on different denoising networks) in Supplementary Material B.

### 3.3 Invariance reconstruction

**FIGURE 5:**
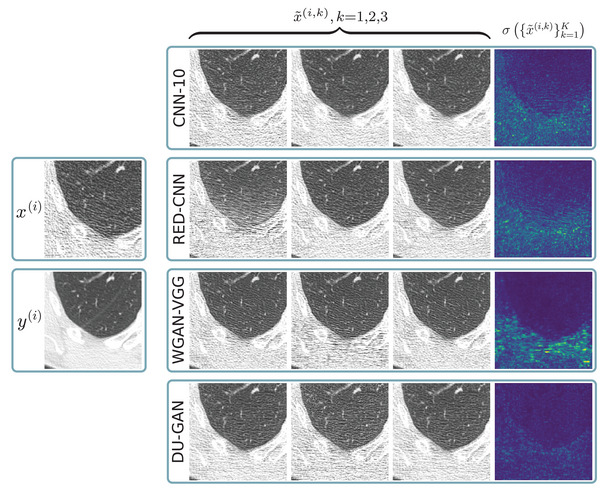
Invariances for a random crop i from the test set. Shown are low‐dose image $x^{\left(i\right)}$, high dose image $y^{\left(i\right)}$ and three reconstructed invariances ${\tilde{x}}^{\left(i,k\right)}$ for each of the four denoising methods. We also show standard deviations $\sigma \left({\left\{{\tilde{x}}^{\left(i,k\right)}\right\}}_{i=1}^{K}\right)$ over K=100 invariances. For CT Images: C=−600HU, W=1500HU, for standard deviations: C=0HU, W=400HU.

Given the procedure described in Section 2.3.3 and shown in Figure 1, we sample 100 invariances for each of the four denoising networks on 1000 random crops of the test set. In Figure 5 we show three invariances for one of those random crops. We find that for all denoising networks sampled invariances mainly differ in terms of noise amplitude and realization. This is expected as the networks see many noise realizations as well as patients of different thickness (influencing the noise level) during the training. These differences in noise structure and amplitude overshadow possible differences in anatomical content between samples. We provide additional results in Supplementary Material B.

### 3.4 Analyzing invariances

#### In the VAE latent space

Next, we analyze sampled invariances in the latent space of the unconditional VAE. To this end we compute for each network and sampled crop i the mean cosine similarity over sampled invariances k=1,2,⋯,K

(9) $$\begin{array}{c} S_{\text{VAE}}^{\left(i\right)}=\frac{1}{K}\sum_{k=1}^{K}\cos\left(z^{\left(i\right)},z^{\left(i,k\right)}\right)\;. \end{array}$$

**FIGURE 6:**
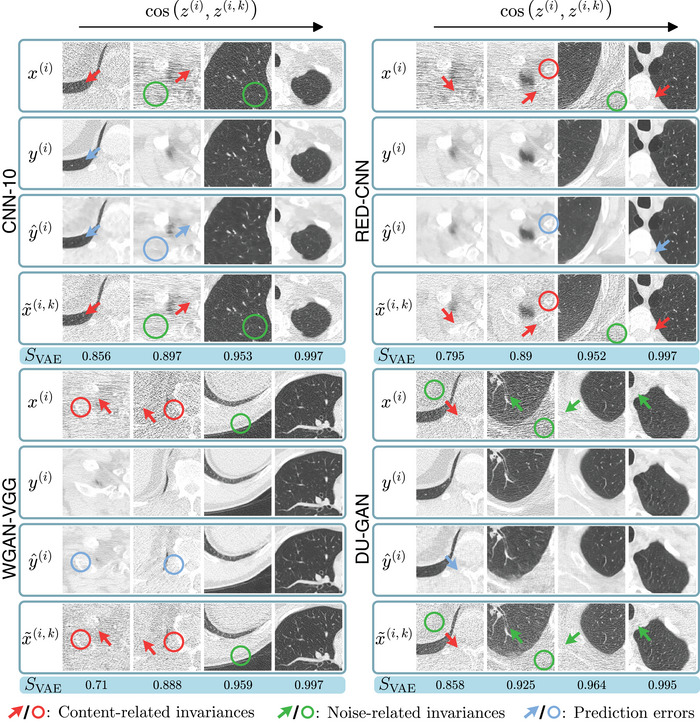
Invariances with increasing $S_{\text{VAE}}$ (left to right), that is, decreasing amount of content‐related invariances as measured in the VAE latent space, for each of the four denoising methods. C=−600HU, W=1500HU.

In Figure 6 we show four crops, corresponding to the {0,1/3,2/3,1} quantiles of the mean similarity $S_{\text{VAE}}^{\left(i\right)}$ over all test samples, for each network. Here we find that for samples with lower $S_{\text{VAE}}^{\left(i\right)}$ (left), most differences between $x^{\left(i\right)}$ and ${\tilde{x}}^{\left(i,k\right)}$ are in terms of anatomical content (red arrows). In contrast, for samples with higher $S^{\left(i\right)}$ (right) anatomical content is similar between $x^{\left(i\right)}$ and ${\tilde{x}}^{\left(i,k\right)}$ and differences are mainly in terms of noise amplitude and realization. This indicates that the latent space of the VAE is indeed dominated by anatomy‐related information and disentangling sampled invariances. We provide further analysis of the VAE latent space in Supplementary Material B.

#### 3.4.1 In the DML latent space

**FIGURE 7:**
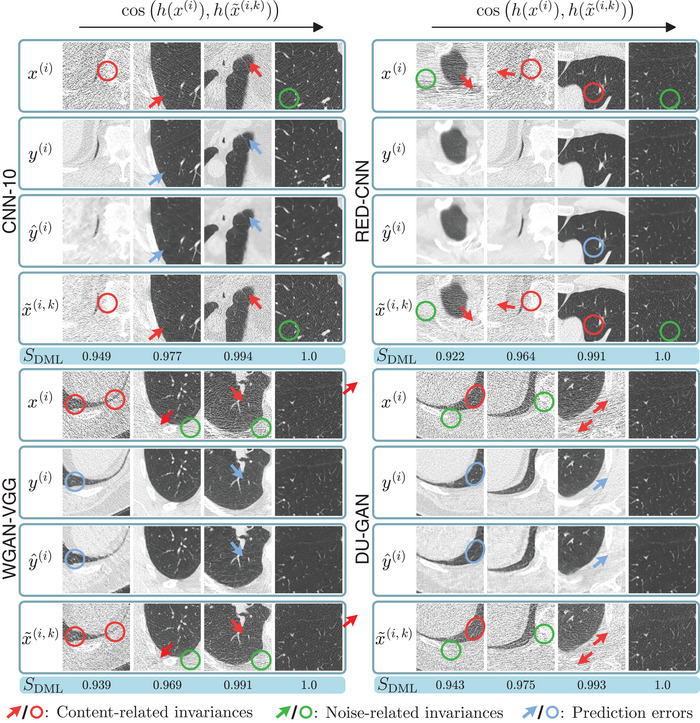
Invariances with increasing $S_{\text{DML}}$ (left to right), that is, decreasing amount of content‐related invariances as measured in the DML embedding space, for each of the four denoising methods. C=−600HU, W=1500HU.

Next, we analyze sampled invariances using the DML‐based embedding. Similar as for the VAE, we measure similarity between $x^{\left(i\right)}$ and ${\tilde{x}}^{\left(i,k\right)}$ using the mean cosine similarity over sampled invariances k=1,2,⋯,K

(10) $$\begin{array}{c} S_{\text{DML}}^{\left(i\right)}=\frac{1}{K}\sum_{k=1}^{K}\cos\left(h\left(x^{\left(i\right)}\right),h\left({\tilde{x}}^{\left(i,k\right)}\right)\right) \end{array}$$

and show four samples with increasing mean similarity $S_{\text{DML}}^{\left(i\right)}$, again corresponding to the {0,1/3,2/3,1} quantiles of the empirical distribution, in Figure 7. For all denoising networks, we find that samples with lower $S_{\text{DML}}^{\left(i\right)}$ (left) exhibit differences in terms of anatomical content (red arrows) while samples with higher $S_{\text{DML}}^{\left(i\right)}$ (right) mainly differ in terms of noise amplitude and realization.

Lastly, we compare the invariances of different denoising networks quantitatively using the pixel‐wise mean absolute difference (MD) between $x^{\left(i\right)}$ and ${\tilde{x}}^{\left(i,k\right)}$ as well as $S_{\text{VAE}}^{\left(i\right)}$ and $S_{\text{DML}}^{\left(i\right)}$ (Table 2). Note that, opposed to $S_{\text{VAE}}$ and $S_{\text{DML}}$, the MD acts in the pixel space and is therefore both a measure of content‐related and noise‐related invariances. We find that quantitatively, the invariances of the denoising networks are very similar, with RED‐CNN showing the highest amount of invariances (higher MD, lower $S_{\text{VAE}}$ and $S_{\text{DML}}$). Upon a statistical analysis using a one‐sided Mann–Whitney U test with Benjamini–Hochberg correction for multiple comparisons, we examine that this finding is significant for most invariance metrics and denoising methods (In Table 2, stars for some method indicate significance levels of the pairwise test that RED‐CNN has more invariances compared to this method).

**TABLE 2:** Quantitative evaluation of invariances using the mean absolute difference (MD), mean cosine similarity in the VAE latent space ($S_{\text{VAE}}$), and mean cosine similarity in the learned embedding space ($S_{\text{DML}}$).

Invariances	MD ↑ noise + content	$S_{\text{VAE}}↓$ content	$S_{\text{DML}}↓$ content
CNN‐10	$182\pm 67^{***}$	$0.978\pm 0.020^{**}$	$0.997\pm 0.004^{***}$
RED‐CNN	191±67	0.976±0.022	0.996±0.007
WGAN‐VGG	$158\pm 64^{***}$	$0.979\pm 0.021^{***}$	0.996±0.006
DU‐GAN	$178\pm 70^{***}$	0.979±0.013	0.997±0.004

*Note*: For MD, higher values imply more noise and content‐related invariances (due to MD being pixel‐wise), for $S_{\mathrm{VAE}}$ and $S_{\mathrm{DML}}$ lower (↓) values imply more anatomical invariances (since they measure similarity of anatomical content between sampled invariances and input images). **Bold** values indicate the denoising method with the highest amount of invariances (↑ MD, $↓S_{\mathrm{VAE}}$, $↓S_{\mathrm{DML}}$). We indicate statistical significance of this finding with 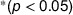, 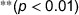, and 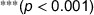.

## 4 DISCUSSION

In this work, we presented a method for reconstructing the invariances of deep learning‐based low‐dose CT image denoising algorithms. Upon reconstructing the invariances of four common denoising networks we found that the sampled invariances mainly differ in terms of noise amplitude and realization, while the anatomical content is largely preserved. This is expected and can be explained by the training procedure of these networks. To answer our initial question of whether LDCT denoising networks are invariant to anatomical structures or other image content, we further proposed two methods to analyze the sampled invariances. Both methods are based on measuring distances between sampled invariances and input images in a lower‐dimensional latent space. Using these methods, we found that all denoising networks are also invariant to anatomical structures to some extent. Quantitatively, the amount of invariances (both noise‐related and content‐related) of different denoising networks are very similar, with RED‐CNN showing the highest amount of invariances both in terms of noise and anatomical structures. In Supplementary Material B we provide additional results for an algorithm that has, by design, more invariances to anatomical structures.

Our method is similar to uncertainty quantification methods such as Monte‐Carlo dropout or moment propagation^51^, ^52^ in that it can improve the interpretability of deep learning‐based methods for medical imaging. However, both approaches provide orthogonal views of the network's behavior. While uncertainty quantification methods provide a measure of the network's confidence in its predictions, our method provides a measure of the network's invariances to the input features. There are many scenarios in which an algorithm can be confident in its prediction but still exhibit invariances to certain input features (e.g., the algorithm analyzed in Supplementary Material B; *Case study: Algorithm with strong invariances by design*). In such cases, our method can provide additional insights into the network's behavior. Lastly, our proposed approaches for analyzing the sampled invariances could also be helpful in analyzing systematic uncertainties quantified using the aforementioned methods, an interesting direction for future work.

## 5 CONCLUSIONS

Our work shows that common LDCT image denoising networks are invariant to certain input features. While these invariances are mostly dominated by noise, all networks investigated in this study are also invariant to anatomical structures to some extent. We believe that developing methods to reconstruct and analyze these invariances is an important step toward interpreting deep learning‐based methods for medical image formation.

Since the presented method is architecture agnostic, several natural extensions of our work come to mind: Promising research directions include (a) evaluating the impact of training data distribution on the invariances of LDCT denoising networks; (b) investigating invariances of other networks for medical imaging including other modalities such as PET and MR; and (c) relating invariances to the similar concept of hallucinations in medical imaging. Lastly, while the sampling of invariances using our method is very fast (≈23ms), future work should reduce the computational complexity of training the two networks, for example by disentangling the invariances in the VAE latent space directly, thus eliminating the need for training of a cINN.

## CONFLICT OF INTEREST STATEMENT

The authors declare no conflicts of interest.

## Supporting information

Supporting Information

## Footnotes

* For example, PubMed (http://pubmed.ncbi.nlm.nih.gov) lists 56 publications in 2023 for the query: (low dose OR low‐dose) AND (Computed Tomography OR CT) AND deep learning AND denoising.

† Code available at https://github.com/eeulig/ldct‐invariances.

‡ Note, that this has the interesting side‐effect that the latent space is already mostly comprised of the invariances of the denoising network as this is exactly the information missing in $\hat{y}$.
