# Supplementary material for: Reconstructing and analyzing the invariances of low‐dose CT image denoising networks
Source: Med Phys. 2024 Sep 30;52(1):188–200. doi: 10.1002/mp.17413 (PMC11700010; doi:10.1002/mp.17413)
Supplement: Supplementary file 1 — Supporting Information [file MP-52-188-s001.pdf]

Appendix: Reconstructing and Analyzing the Invariances of Low-Dose CT Image Denoising Networks

A. Training details

A.1. Denoising networks

For all denoising methods, we determine the optimal hyperparameters via Bayesian optimization on the *Low Dose CT Image and Projection Dataset*[31]. For more details we refer the reader to Eulig et al., 2024[32] and list the hyperparameters here for reproducibility: **CNN-10** was trained on patches of size 92×92 px with a mini-batch size of 68, and using the Adam optimizer with learning rate 1.6×10^{-4}. **RED-CNN** was trained on patches of size 128×128 px, with a mini-batch size of 73, and using the Adam optimizer with learning rate 9.6×10^{-5}. **WGAN-VGG** was trained on patches of size 77×77 px, with a mini-batch size of 77, and using the Adam optimizer with learning rate 7.1×10^{-5} and $\beta_1 = 0.33$. The perceptual loss is evaluated after the last convolutional layer of a pretrained VGG-19 network and weighted with $\lambda_{\text{perc}} = 0.689$. **DU-GAN** was trained on patches of size 128×128 px, with a mini-batch size of 92, and using the Adam optimizer with learning rate 1.2×10^{-5} and $\beta_1 = 0.65$. As weighting parameters for the generator loss we used $\lambda_{\text{img}} = 1.0, \lambda_{\text{grd}} = 27.8, \lambda_{\text{adv}} = 0.1$. We use the same CutMix regularization as described in the original publication.

A.2. Conditional variational autoencoder

Our conditional autoencoder uses an ImageNet-pretrained ResNet-50[38] as encoder that predicts $\mu^{(i)}, \sigma^{(i)} \in \mathbb{R}^M$ with $M = 256$. Latent variables are mapped to image space using a decoder based on BigGAN[39]. To improve reconstruction quality we use a mixture of pixelwise, perceptual, and adversarial loss for the reconstruction term $\mathcal{L}_{\text{rec}}$ in Eq. (4):

$$\begin{aligned}\mathcal{L}_{\text{rec}} &= \mathcal{L}_{\text{adv}} + \lambda_{\text{pix}}\mathcal{L}_{\text{pix}} + \lambda_{\text{perc}}\mathcal{L}_{\text{perc}} \\ &= \mathbb{E}_i\left[-C\left(\hat{x}^{(i)}|\hat{y}^{(i)}\right) + \lambda_{\text{pix}}|x^{(i)} - \hat{x}^{(i)}| + \lambda_{\text{perc}}\frac{1}{L}\sum_{l=1}^{L}|\phi_l^{\text{VGG}}(x^{(i)}) - \phi_l^{\text{VGG}}(\hat{x}^{(i)})|\right], \quad (11)\end{aligned}$$

with $\lambda_{\text{pix}} = 1.2 \times 10^{-3}, \lambda_{\text{perc}} = 0.12$ determined through a randomized search. The perceptual loss is evaluated before each of the first $L = 4$ max-pooling operations of an

ImageNet-pretrained VGG-16 ϕ^{VGG}. The adversarial loss is evaluated using a critic with six blocks where each block consists of $\mathrm{Conv2D}(f_{\mathrm{in}}, f_{\mathrm{out}}, \mathrm{kernel\_size} = 4, \mathrm{stride} = 2) \rightarrow \mathrm{InstanceNorm} \rightarrow \mathrm{LeakyReLU}(\mathrm{negative\_slope} = 0.2)$ with $f_{\mathrm{in}} = 2, 64, 128, 256, 512, 512$ and $f_{\mathrm{out}} = 64, 128, 256, 512, 512, 512$. Lastly, a single 1×1 convolution followed by a fully-connected layer $\mathrm{Linear}(16, 1)$ is used to map to a scalar output.

Both generator and critic are trained with a batch size of 64 using an Adam optimizer with $\beta_1 = 0.5$ and a learning rate of 1×10^{-5} for the generator and 4×10^{-5} for the critic.

A.3. Conditional invertible neural network

The cINN t_ξ consists of 12 invertible blocks, each of which consists of an affine coupling layer[41], ActNorm[43], and a shuffling layer. The output of the denoising network is first embedded using a shallow CNN and then concatenated to each of the fully connected networks in the coupling layers to realize the conditioning. Since our architectural design closely follows the design of the cINN in Rombach et al., 2021[14] we refer the reader to the original publication for further details.

We train the cINN with the loss function in Eq. (6) using the Adam optimizer and a learning rate of 1×10^{-5} and a mini-batch size of 128.

A.4. Learned embedding

As DML model h_ν we use the feature encoder of an ImageNet-pretrained ResNet-50 with a single fully-connected layer after the average pooling, mapping to the 32-dimensional embedding space. All embeddings are ℓ_2 normalized. During training, we sample triplets $(a, p, n) := (x^{(i)}, \tilde{x}^{(i,k)}, \tilde{x}^{(j,l)})$ using the online version of the semi-hard triplet mining strategy[45], *i.e.*, for all positives and negatives in a batch we only use triplets for which the negative is further away from the anchor than the positive but violates the margin α:

$$d(h(a), h(p)) < d(h(a), h(n)) < d(h(a), h(p)) + \alpha \,. \tag{12}$$

As most related works, we use the squared euclidian distance $d(x, y) = \|x - y\|_2^2$ as distance metric during training. Note that for ℓ_2-normalized embeddings, using the cosine similarity Eq. (10) for analyzing sampled invariances does not alter the ranking of similar/dissimilar

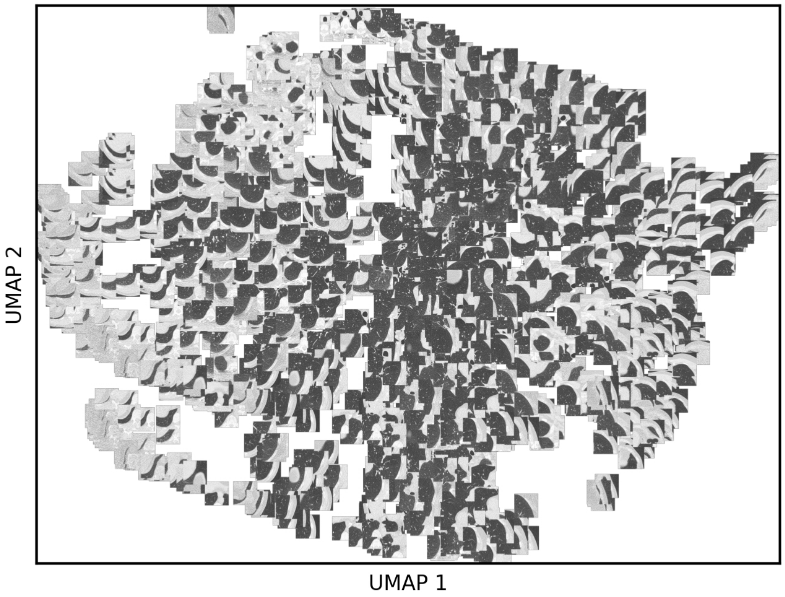

Figure B.1: UMAP visualization of the 256-dimensional VAE latent space for 1,000 samples $x^{(i)}$ from the test dataset, encoded using the unconditional VAE.

samples shown in Fig. 7 since

$$\|x - y\|_2^2 = 2 - 2\cos(x, y) \quad \forall \|x\|_2 = \|y\|_2 = 1\,. \tag{13}$$

We train the model using the Adam optimizer with a learning rate of 1×10^{-4} and a batch size of 128. The margin α is set to 0.2.

B. Additional results

VAE latent space To further test our hypothesis that the VAE latent space is dominated by anatomical information, we visualize its latent space in Fig. B.1. This is done by sampling 1,000 images from the test dataset, predicting the mean using the VAE encoder and applying UMAP[53] to reduce the 256 latent dimensions down to two. Here we find that similar anatomical structures and regions are, independently of their noise structure and realization, embedded close together in the latent space.

Distributions of $S_{\mathbf{VAE}}$ and $S_{\mathbf{DML}}$ We show violinplots of the cosine similarities S_{VAE} and S_{DML} for all 1000 random samples from the test set in Fig. B.2.

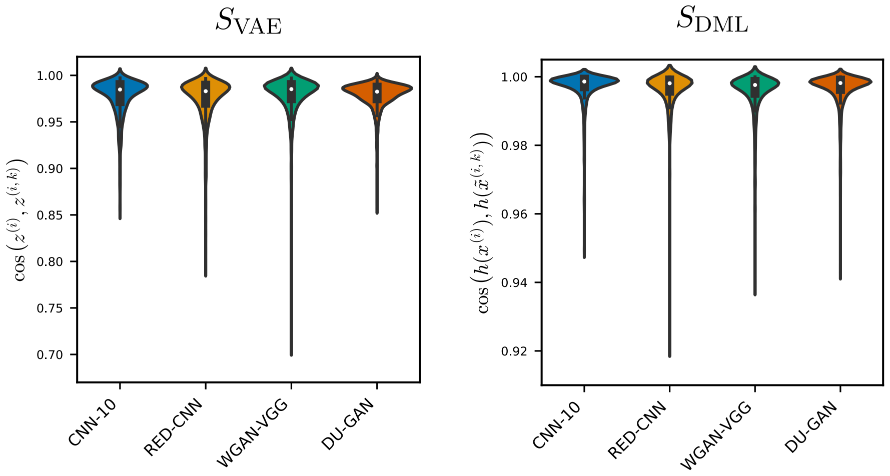

Figure B.2: Violinplots of the cosine similarities S_{VAE} and S_{DML} for all 1000 random samples from the test set.

Conditional VAE reconstructions The ability of the conditional VAE to faithfully reconstruct the input is to some extent dependent on the conditioning $\hat{y} = f_\theta(x)$ and therefore on the denoising network f_θ. We show reconstruction capabilities for all four conditional VAEs (conditioned on the four denoising networks, respectively) as well as for an unconditonal VAE in Fig. B.3. We find that while there are, as expected, small differences in the reconstructions, all conditional VAEs are able to reconstruct the low dose images very well and are able to capture all anatomical structures. In particular, all conditional VAEs perform much better than the unconditional VAE used in previous work[30].

Case study: Algorithm with strong invariances by design Since all four networks investigated in our study showed relatively little invariances to anatomical structures, we conduct a case study where we reconstruct the invariances of a dummy algorithm that has strong localized invariances by design. Let $M \in \{0,1\}^{n\times m}$ be a binary mask of same size as the input images $x^{(i)} \in \mathbb{R}^{n\times m}$ then the output image $\hat{y}^{(i)} \in \mathbb{R}^{n\times m}$ of this algorithm is given as

$$\hat{y}^{(i)} = x^{(i)} \odot (1 - M)\,, \tag{14}$$

with $\odot$ denoting the element-wise product, *i.e.* all pixels in $x^{(i)}$ for which M is one are set to zero. In our experiments we use a mask M that is a square of size 30×30 pixels centered in the image and denote the resulting algorithm as **Center-Inv**. We expect this algorithm to be, by design, invariant w.r.t. input information within this center square but not invariant to

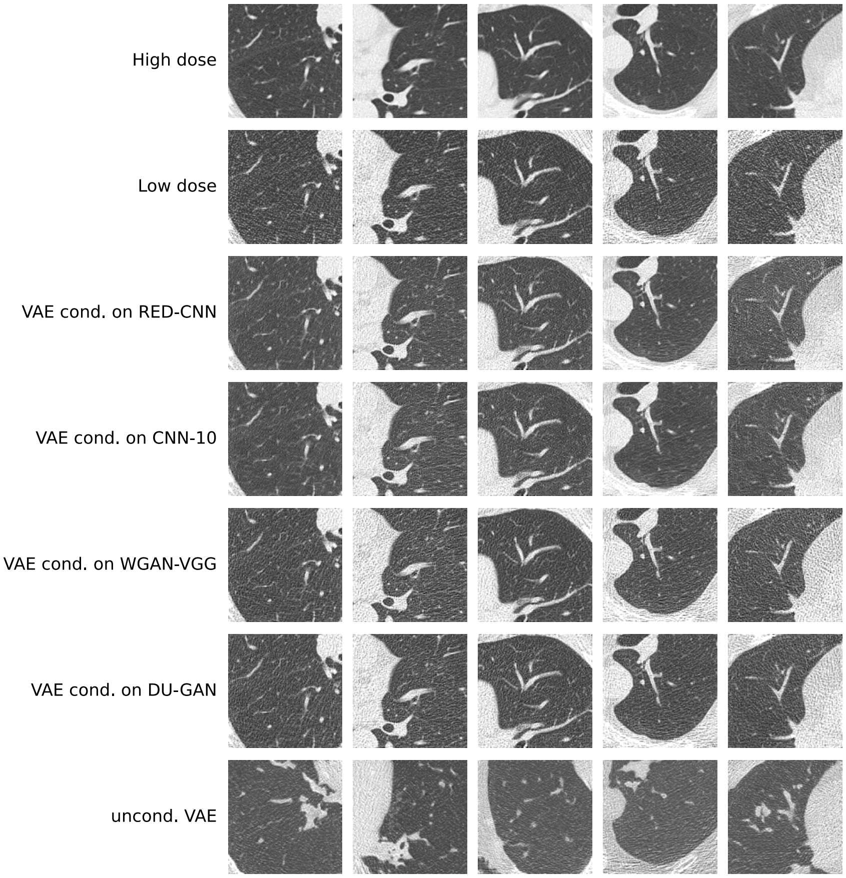

Figure B.3: High dose, low dose, and VAE reconstructions for all four conditional VAEs used in this work (conditioned on the four denoising networks, respectively). Additionally, we show the reconstructions of an unconditional VAE (as used in previous work[30]) for comparison. $C = -600\,\mathrm{HU}$, $W = 1500\,\mathrm{HU}$.

anything outside the square. For this algorithm we train an additional conditional VAE and cINN as described in App. A.2. and A.3.. We show one random example of reconstructed invariances for Center-Inv in Fig. B.4. We find that our method is able to successfully reconstruct the invariances within the center square while showing close-to-zero invariances outside the square. Within the center square, differences between sampled invariances are larger than they are for the other four algorithms, as expected (Fig. B.4; rightmost column). We also perform a quantitative evaluation using the mean absolute difference (MD), mean cosine similarity in the VAE latent space (S_{VAE}), and mean cosine similarity in the learned embedding space (S_{DML}), similar to the evaluation in Sec. III.D. and show the results in Tab. B.1. We find that Center-Inv has significantly more anatomical invariances compared to the other algorithms as measured by the S_{VAE} and S_{DML}. Furthermore, we find that the mean absolute difference (MD) between reconstructed invariances and input images is

Table B.1: Quantitative evaluation of invariances using the mean absolute difference (MD), mean cosine similarity in the VAE latent space (S_{VAE}), and mean cosine similarity in the learned embedding space (S_{DML}). The first four rows are identical to Tab. 2 and are shown for comparison.

Invariances	MD ↑<br>noise + content	S_{VAE} ↓<br>content	S_{DML} ↓<br>content
CNN-10	182 ± 67	0.978 ± 0.020	0.997 ± 0.004
RED-CNN	$\mathbf{191 \pm 67}$	0.976 ± 0.022	0.996 ± 0.007
WGAN-VGG	158 ± 64	0.979 ± 0.021	0.996 ± 0.006
DU-GAN	178 ± 70	0.979 ± 0.013	0.997 ± 0.004
Center-Inv	46 ± 17	$\mathbf{0.960 \pm 0.032}$	$\mathbf{0.983 \pm 0.041}$

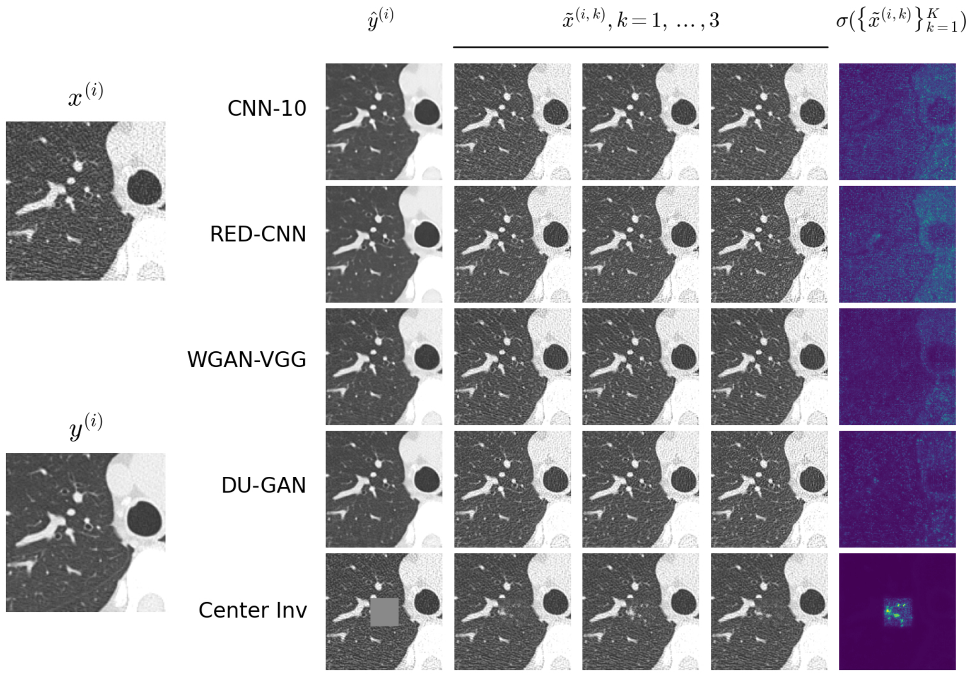

Figure B.4: Three reconstructed invariances for a random sample from the test set. For each of the methods we also show the denoised image $\hat{y}$ and the standard deviation map over all $K = 100$ sampled invariances. For CT Images: $C = -600$ HU, $W = 1500$ HU, for standard deviations: $C = 0$ HU, $W = 300$ HU.

lower for Center-Inv than for the other algorithms. This can be explained by our method successfully predicting almost zero invariances for all pixels outside the mask which account for $\approx 94.5\%$ of all pixels in the image.

Reconstructed invariances We present additional invariances reconstructed for six random samples from the test set in Figs. B.5 to B.10.

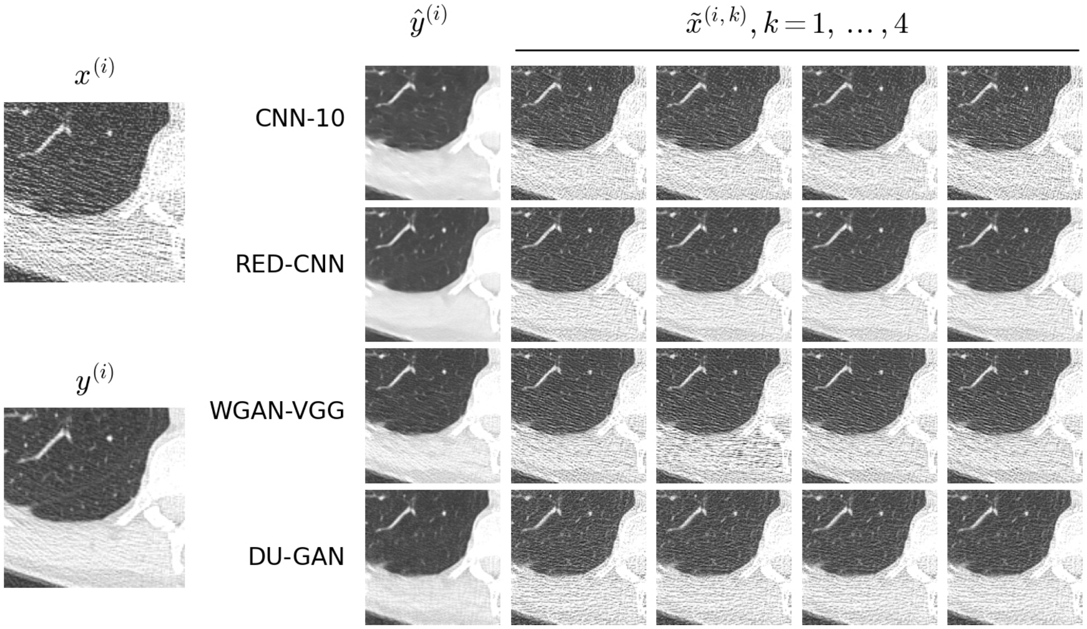

Figure B.5: Four reconstructed invariances for a random sample from the test set. For each of the methods we also show the denoised image $\hat{y}$. $C = -600\,\mathrm{HU}$, $W = 1500\,\mathrm{HU}$.

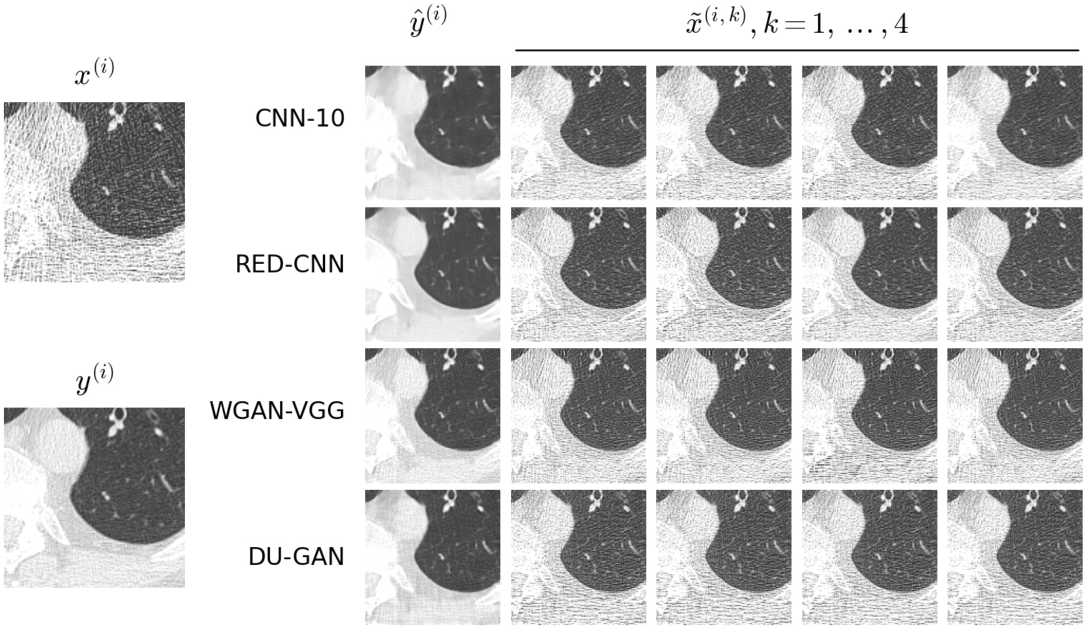

Figure B.6: Four reconstructed invariances for a random sample from the test set. For each of the methods we also show the denoised image $\hat{y}$. $C = -600\,\mathrm{HU}$, $W = 1500\,\mathrm{HU}$.

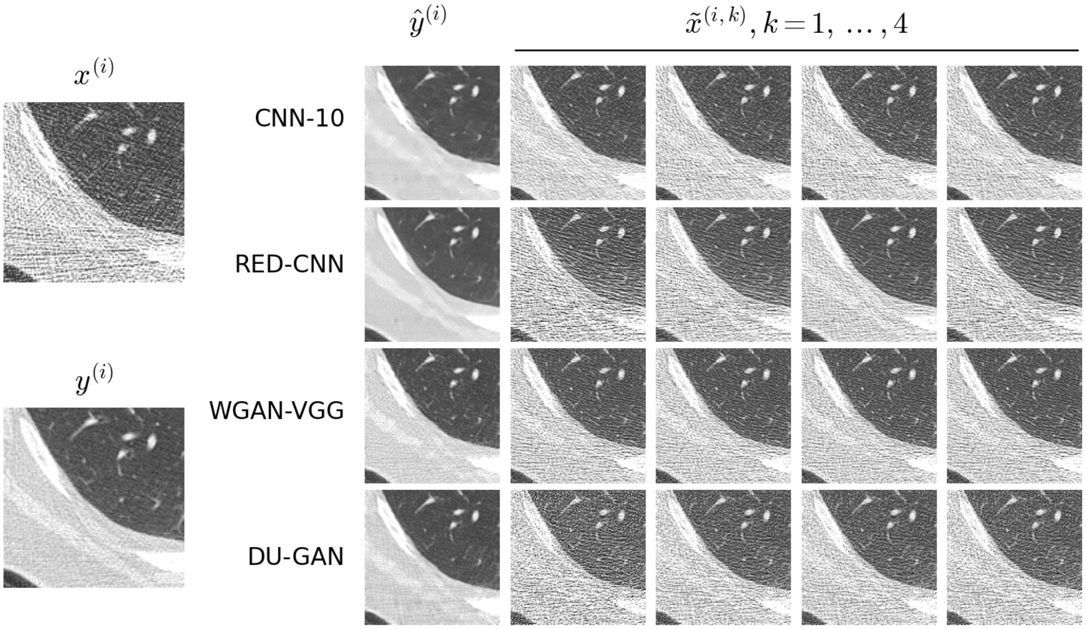

Figure B.7: Four reconstructed invariances for a random sample from the test set. For each of the methods we also show the denoised image $\hat{y}$. $C = -600\,\mathrm{HU}$, $W = 1500\,\mathrm{HU}$.

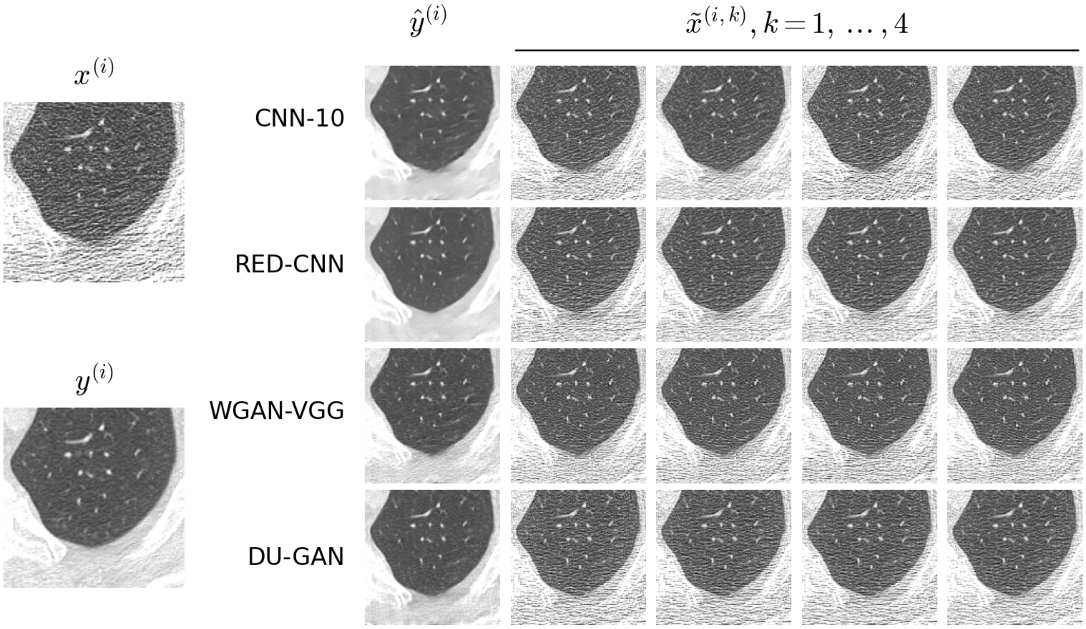

Figure B.8: Four reconstructed invariances for a random sample from the test set. For each of the methods we also show the denoised image $\hat{y}$. $C = -600\,\mathrm{HU}$, $W = 1500\,\mathrm{HU}$.

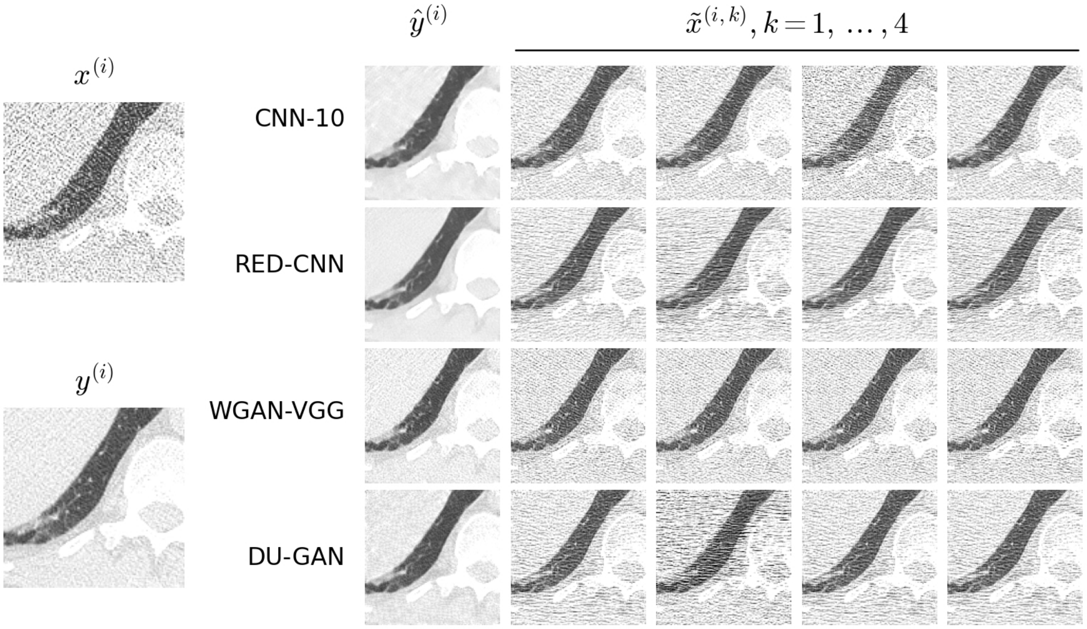

Figure B.9: Four reconstructed invariances for a random sample from the test set. For each of the methods we also show the denoised image $\hat{y}$. $C = -600\,\mathrm{HU}$, $W = 1500\,\mathrm{HU}$.

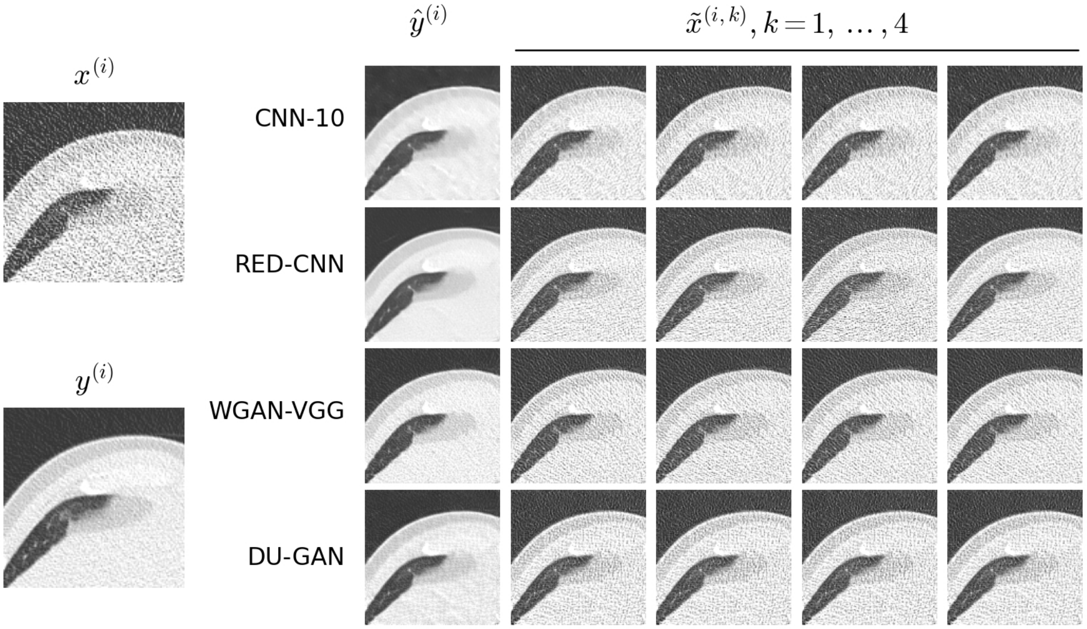

Figure B.10: Four reconstructed invariances for a random sample from the test set. For each of the methods we also show the denoised image $\hat{y}$. $C = -600\,\mathrm{HU}$, $W = 1500\,\mathrm{HU}$.
